# Entropy Production on the Gravity-Driven Flow with Free Surface Down an Inclined Plane Subjected to Constant Temperature

**DOI:** 10.3390/e20040293

**Published:** 2018-04-17

**Authors:** Jaesung Lee

**Affiliations:** Department of Chemical and Environmental Technology, Inha Technical College, Incheon 22212, Korea; JLee@inhatc.ac.kr; Tel.: +82-32-870-2274

**Keywords:** falling film, entropy, long-wave approximation

## Abstract

The long-wave approximation of a falling film down an inclined plane with constant temperature is used to investigate the volumetric averaged entropy production. The velocity and temperature fields are numerically computed by the evolution equation at the deformable free interface. The dynamics of a falling film have an important role in the entropy production. When the layer shows an unstable evolution, the entropy production by fluid friction is much larger than that of the film with a stable flat interface. As the heat transfers actively from the free surface to the ambient air, the temperature gradient inside flowing films becomes large and the entropy generation by heat transfer increases. The contribution of fluid friction on the volumetric averaged entropy production is larger than that of heat transfer at moderate and high viscous dissipation parameters.

## 1. Introduction

Gravity-driven flows have attracted a great deal of attention in industrial processes such as coating processes, in which the instability of the flowing film is highly undesirable. Therefore, the stability of a falling film down an inclined plane has been extensively studied. The linear stability analysis was initiated by Yih [1], who used lubrication theory with a small parameter as the ratio of initial film thickness to the characteristic wavelength. He derived and solved the Orr–Sommerfeld equations to determine the critical Reynolds number above which the liquid film is unstable. Benny [2] and Gjevik [3] expanded to the nonlinear regime and derived a nonlinear evolution equation for the film height. Lin [4] investigated a weakly nonlinear analysis to study the side-band stability near neutral curve. Chang [5] used the Hopf bifurcation theory to investigate the periodic waves near the upper neutral curves. The isothermal solitary waves were numerically studied by Pumar et al. [6] and Nakaya [7]. They showed that there are branch multiplicity and turning points depending on the Reynolds number to decide the existence of solitary waves. The dynamics of thin film flows have different characteristics depending on flow rates [8,9,10]. If a critical flow rate is the flow rate when the maximum surface velocity is equal to the wave speed, the fluid below the critical flow rates has the highest surface velocity at the crest. However, above the critical flow rate, circulating waves are found due to the circulating eddy on the interfacial side, and this has an important effect on the interfacial heat and mass transfer [11]. This circulating eddy comes from the splits and movements of stagnation points, and increases the viscous dissipation. Dietze et al. carried out numerical studies on three-dimensional flow structures for falling liquid films and compared with experimental works [12]. They found that the liquid layer can be developed as a thin residual layer without capillary waves.

When heat transfers to the film from the bottom plane, the volatile liquid film has instabilities at the free surface such as thermocapillarity and vapor recoil. Thermocapillarity is induced by the temperature gradient of surface tension at the free surface and vapor recoil occurs due to the difference of evaporation rate on an evaporating liquid surface. The long-wave approach was also used to study the linear and nonlinear stability analysis of a liquid film on heated or cooled planes. The destabilization with thermocapillarity was shown by Kelly et al. [13] and the instability of vapor recoil was studied by Bankoff [14] and Burelbach et al. [15]. The evolution equations of heated falling films was extensively studied by Joo et al. [16,17]. Rietz et al. conducted experiments on surface topology in regular three-dimensional falling films with heat flux conditions [18]. They investigated the interactions between hydrodynamics and thermocapillary forces and showed the formation of rivulets and film thinning.

The motion of fluids is influenced by the boundary conditions at the plane. The constant temperature condition can be considered as a plane of infinite thermal conductivity and heat capacity, while the constant heat flux condition corresponds to a poorly conducting plane. The comparison between the two boundary conditions was investigated for turbulent thermal convection [19,20]. Trevelyan et al. [10] constructed bifurcation diagrams for travelling solitary waves subjected to two imposed boundary conditions of a heat flux and a specified temperature, assuming that the surface tension was expressed as a temperature-dependent property. They derived the critical conditions for the onset of the instability and the evolution equation for the deformable free interface for large Peclet numbers. They computed the solitary waves depending on the boundary conditions and validated the long-wave assumption to describe the recirculation zone below a solitary hump.

The entropy production of a Newtonian laminar falling film along an inclined plane was investigated by Saouli and Aiboud-Saouli [21], where the flat interface was assumed to be free and adiabatic and constant heat flux condition was applied to the bottom plane. The second law analysis of a non-Newtonian laminar falling film down an inclined plane was studied by Gorla and Pratt [22], where the power-law model was used and the constant heat flux condition was also used. They analytically obtained the temperature fields with the separation of variables in two-dimensional space, and showed that the entropy production rate and the irreversibility ratio in the transverse direction decreased. Sahin [23,24] obtained the entropy generation rate of turbulent flows through a circular pipe corresponding to constant wall temperature and constant heat flux, where viscosity was dependent on temperature. He showed that viscosity variation with temperature allows a significant contribution to the entropy production and pumping power. As an application for absorber optimization, an absorptive falling film over a cooled horizontal tube absorber was exemplified to determine the minimum mass flow rates [25].

The purpose of this study is to address the viscous dissipation effects in a non-volatile liquid film flowing down an inclined heated plane with constant temperature. The novelty of this paper lies in providing a way to compute the entropy production rate of falling films with a deformable free interface. The key is to obtain the velocity and temperature fields from the evolution equation for the perturbed free surface, where the long-wave expansion approach is used. The evolution of film height with time is solved by the method of lines with the FFT (Fast Fourier transform).

## 2. Formulation

**Figure 1:**
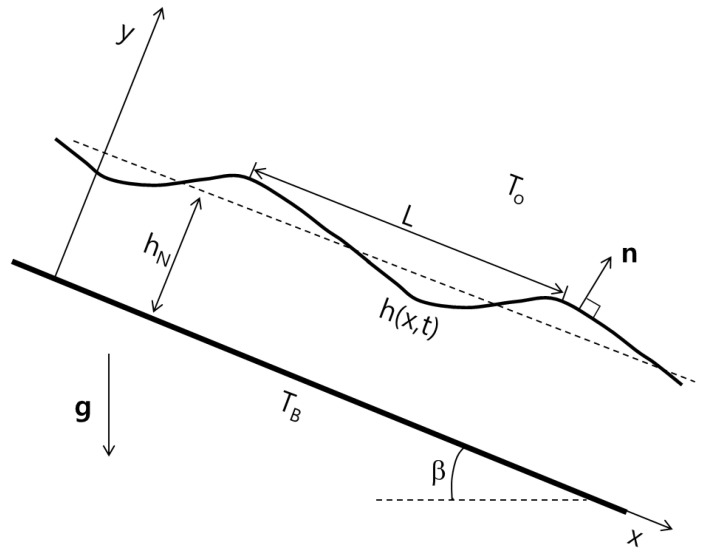
The physical configuration.

We consider the non-isothermal Newtonian flow down an inclined plane with an inclination angle β with constant density ρ, viscosity μ, and thermal conductivity *k*, where the velocity field u is defined by (u,v) in two-dimensional space. The layer is assumed to be an incompressible and non-volatile liquid bounded by an ambient gas with pressure $p_{0}$ and temperature $T_{0}$, where the physical properties of the liquid phase are considered much more than those of the gas phase. As shown in Figure 1, the normal coordinate *y* is taken to be zero on the plane and the deformable free surface is expressed by y=h(x,t), where *x* is the lateral coordinate and *t* is time. The temperature at the bottom wall keeps at a constant value of $T_{B}$.

### 2.1. Governing Equations and Boundary Conditions

The continuity equation corresponding to u=(u,v) is

(1) $$u_{x}+v_{y}=0.$$

Hereafter, the subscript indicates the derivative with respect to it (i.e., $u_{x}=\partial u/\partial x$). The equations of motion in two-dimensional space are given by

(2) $$\begin{array}{cc} \rho \left(u_{t}+uu_{x}+vu_{y}\right) & =-p_{x}+\mu \left(u_{xx}+u_{yy}\right)+\rho g\sin\beta , \end{array}$$

(3) $$\begin{array}{cc} \rho \left(v_{t}+uv_{x}+vv_{y}\right) & =-p_{y}+\mu \left(v_{xx}+v_{yy}\right)-\rho g\cos\beta , \end{array}$$

where *g* is a gravitational acceleration constant. The energy equation is expressed by

(4) $$\begin{array}{cc} T_{t}+uT_{x}+vT_{y} & =\kappa \left(T_{xx}+T_{yy}\right), \end{array}$$

where κ is the thermal diffusivity defined by $k/\rho C_{p}$ and $C_{p}$ is the heat capacity.

The boundary conditions at the bottom plane are

(5) $$\begin{array}{c} u=0\;\;\mathrm{and}\;\;T=T_{B}. \end{array}$$

At the deformable free surface, the jump normal and shear stress conditions [16] are, respectively,

(6) $$\begin{array}{cc} p-p_{0}-\frac{2\mu }{\left(1+h_{x}^{2}\right)}\left(h_{x}^{2}u_{x}-h_{x}\left(u_{y}+v_{x}\right)+v_{y}\right) & =-\frac{\sigma h_{xx}}{{\left(1+h_{x}^{2}\right)}^{3/2}}, \end{array}$$

(7) $$\begin{array}{cc} \left(u_{y}+v_{x}\right)\left(1-h_{x}^{2}\right)+2h_{x}\left(v_{y}-u_{x}\right) & =0, \end{array}$$

where σ is a surface tension. Note that the normal vector $n=\left(-h_{x},1\right)/\left(1+h_{x}^{2}\right)$ is chosen as a vector pointing from the liquid into the ambient air. Applying the Newton’s cooling law to the interface gives

(8) $$\begin{array}{cc} \frac{k}{1+h_{x}^{2}}\left(h_{x}T_{x}-T_{y}\right) & =h_{N}\left(T-T_{\infty }\right), \end{array}$$

where $h_{T}$ is the heat transfer coefficient of the ambient air. The kinematic condition at the deformable free surface gives

(9) $$\begin{array}{c} h_{t}+uh_{x}-v=0. \end{array}$$

In order to get the dimensionless governing equations, the following relations are used:

(10) $$\begin{array}{cc} {\left(x,y,h\right)}^{*}= & \left(\frac{x}{L},\frac{y}{\epsilon L},\frac{h}{\epsilon L}\right),\;\;t^{*}=\frac{t}{L/U},\;\;{\left(u,v\right)}^{*}=\left(\frac{u}{U},\frac{v}{\epsilon U}\right),\;\;\\ & p^{*}=\frac{p}{\rho U^{2}},\;\;\;\mathrm{and}\;\;\;T^{*}=\frac{T-T_{o}}{T_{B}-T_{o}}. \end{array}$$

Here, a long-wave parameter ϵ is defined by $h_{N}/L$, *L* is the characteristic length, $h_{N}$ is the Nusselt flat film depth, *U* is the maximum velocity of a flat film at the free surface defined by $\rho g\sin\beta h_{N}^{2}/2\mu$, and $T_{o}$ is the reference temperature far from the interface in the ambient fluid. The temperature is scaled by the total temperature difference $\Delta T=T_{B}-T_{o}$. The superscript ∗ is neglected for simplicity.

The dimensionless governing equations are

(11) $$\begin{array}{cc} u_{x}+v_{y} & =0, \end{array}$$

(12) $$\begin{array}{cc} \epsilon \left(u_{t}+uu_{x}+vu_{y}\right) & =-\epsilon p_{x}+\frac{1}{Re}\left(\epsilon^{2}u_{xx}+u_{yy}\right)+\frac{\sin\beta }{Fr^{2}}, \end{array}$$

(13) $$\begin{array}{cc} \epsilon^{2}\left(v_{t}+uv_{x}+vv_{y}\right) & =-p_{y}+\frac{\epsilon }{Re}\left(\epsilon^{2}v_{xx}+v_{yy}\right)-\frac{\cos\beta }{Fr^{2}}, \end{array}$$

(14) $$\begin{array}{cc} \epsilon \left(T_{t}+uT_{x}+vT_{y}\right) & =\frac{1}{Pe}\left(\epsilon^{2}T_{xx}+T_{yy}\right). \end{array}$$

Here, the Reynolods number Re, the Froude number Fr, and the Peclet number Pe are defined by

(15) $$Re=\frac{\rho Uh_{N}}{\mu },\;\;Fr=\frac{U}{\sqrt{gh_{N}}},\;\;\mathrm{and}\;\;Pe=\frac{Uh_{N}}{\kappa }.$$

The boundary conditions on the solid plane are

(16) $$\begin{array}{c} u=0\;\;\mathrm{and}\;\;T=1. \end{array}$$

The boundary conditions at the deformable free surface can be arranged as

(17) $$\begin{array}{cc} p-p_{0}-\frac{2\epsilon }{Re(1+\epsilon^{2}h_{x}^{2})}\left(\epsilon^{2}h_{x}^{2}u_{x}-h_{x}\left(u_{y}+\epsilon v_{x}\right)+v_{y}\right) & =-\frac{Wh_{xx}}{{\left(1+\epsilon^{2}h_{x}^{2}\right)}^{3/2}}, \end{array}$$

(18) $$\begin{array}{cc} \left(u_{y}+\epsilon^{2}v_{x}\right)\left(1-\epsilon^{2}h_{x}^{2}\right)+2\epsilon^{2}h_{x}\left(v_{y}-u_{x}\right) & =0, \end{array}$$

(19) $$\begin{array}{cc} \frac{1}{1+\epsilon^{2}h_{x}^{2}}\left(\epsilon^{2}h_{x}T_{x}-T_{y}\right) & =BiT, \end{array}$$

where the Weber number We and the Biot number Bi are denoted by

(20) $$We=\frac{\sigma }{\rho U^{2}h_{N}}\;\;\mathrm{and}\;\;Bi=\frac{h_{T}h_{N}}{k}.$$

Note that the modified Weber number $W=\epsilon^{2}We$ and the Weber number We is assumed by $O(\epsilon^{-2})$. Here, As the Bi approaches zero, the film can be considered as an adiabatic liquid film. The evolution equation to describe the motion of film thickness can be obtained by the kinematic condition,

(21) $$\begin{array}{c} h_{t}+uh_{x}-v=0. \end{array}$$

### 2.2. Perturbed Solutions

Let $u=u^{\left(0\right)}+\epsilon u^{\left(1\right)}$, $p=p^{\left(0\right)}+\epsilon p^{\left(1\right)}$, and $T=T^{\left(0\right)}+\epsilon T^{\left(1\right)}$. Substituting these relations into the governing Equations (11)–(14) and the boundary conditions (16)–(19), the solutions for the leading order can be obtained by

(22) $$\begin{array}{cc} u^{\left(0\right)} & =\frac{Re\sin\beta h^{2}}{Fr^{2}}\left[\frac{y}{h}-\frac{1}{2}\left(\frac{y}{h}\right)\right]=2\left(hy-\frac{y^{2}}{2}\right), \end{array}$$

(23) $$\begin{array}{cc} v^{\left(0\right)} & =-\frac{Re\sin\beta }{2Fr^{2}}h_{x}y^{2}=-h_{x}y^{2}, \end{array}$$

(24) $$\begin{array}{cc} p^{\left(0\right)} & =\frac{2\cot\beta }{Re}\left(h-y\right)+p_{0}-Wh_{xx}, \end{array}$$

(25) $$\begin{array}{cc} T^{\left(0\right)} & =1-\frac{Bi}{1+Bih}y. \end{array}$$

It can be easily checed that the solution of $u^{\left(0\right)}$ shows the parabolic velocity profile and $v^{\left(0\right)}$ with the flat interface is vanished inside the liquid layer. Note that the relation of $Re\sin\beta =2Fr^{2}$ is used. In the adiabatic case (i.e., Bi→0), the leading order solution of the temperature field is reduced to $T^{\left(0\right)}=1$. This represents that the whole liquid layer at steady state has the same temperature as the bottom plate because there is no heat removal from the interface to the ambient fluid. When Bi→∞ (i.e., heat transfer works actively into the gas phase), the leading order solution of temperature field is reduced to $T^{\left(0\right)}=1-y/h$ and the temperature at the interface is always $T^{\left(0\right)}=0$, irrespective of the interface shape. Substituting the solutions of velocity fields Equations (22) and (23) into Equation (21), the kinematic condition for the leading order yields

(26) $$h_{t}=-2h^{2}h_{x}.$$

The governing equations for the first order of O(ϵ) can be recast into

(27) $$\begin{array}{cc} u_{x}^{\left(1\right)}+v_{y}^{\left(1\right)} & =0, \end{array}$$

(28) $$\begin{array}{cc} u_{t}^{\left(0\right)}+u^{\left(0\right)}u_{x}^{\left(0\right)}+v^{\left(0\right)}u_{y}^{\left(0\right)} & =-p_{x}^{\left(0\right)}+\frac{1}{Re}u_{yy}^{\left(1\right)}, \end{array}$$

(29) $$\begin{array}{cc} 0 & =-p_{y}^{\left(1\right)}+\frac{1}{Re}v_{yy}^{\left(0\right)}, \end{array}$$

(30) $$\begin{array}{cc} T_{t}^{\left(0\right)}+u^{\left(0\right)}T_{x}^{\left(0\right)}+v^{\left(0\right)}T_{y}^{\left(0\right)} & =\frac{1}{Pe}T_{yy}^{\left(1\right)}. \end{array}$$

The boundary conditions at the plane are

(31) $$\begin{array}{c} u^{\left(1\right)}=0\;\;\mathrm{and}\;\;T^{\left(1\right)}=0. \end{array}$$

At the deformable free surface, the boundary conditions are

(32) $$\begin{array}{cc} p^{\left(1\right)}-\frac{2}{Re}\left(-h_{x}u_{y}^{\left(0\right)}+v_{y}^{\left(0\right)}\right) & =0, \end{array}$$

(33) $$\begin{array}{cc} u_{y}^{\left(1\right)} & =0, \end{array}$$

(34) $$\begin{array}{cc} T_{y}^{\left(1\right)} & =0. \end{array}$$

$u^{\left(1\right)}$ can be obtained by integrating Equation (28) with respect to *y* twice and using the boundary conditions of Equations (31) and (33). Substituting the solution into Equation (27) with the no-slip boundary condition Equation (31) yields the solution of $v^{\left(1\right)}$. $p^{\left(1\right)}$ can be solved by integrating Equation (29) and applying boundary condition Equation (32). Finally, the temperature field can be directly acquired by Equations (30) and (34). Then, the solutions for O(ϵ) can be arranged as

(35) $$\begin{array}{c} \begin{array}{cc} u^{\left(1\right)} & =Re\left(\frac{hh_{x}}{6}y^{4}-\frac{2h^{2}h_{x}}{3}y^{3}+\frac{\Phi }{2}y^{2}+\frac{4h^{4}h_{x}}{3}y-\Phi hy\right), \end{array} \end{array}$$

(36) $$\begin{array}{cc} v^{\left(1\right)} & =\;Re\;\left[\;-\;\left(\;\frac{hh_{xx}}{30}\;+\;\frac{h_{x}^{2}}{30}\;\right)\;y^{5}\;+\;\left(\;\frac{h^{2}h_{xx}}{6}\;+\;\frac{hh_{x}^{2}}{3}\;\right)\;y^{4}\;-\;\frac{\Phi_{x}}{6}y^{3}\right.\\ & \;\;\;\left.\;-\;\left(\;\frac{2h^{4}h_{xx}}{3}\;+\;\frac{8h^{3}h_{x}^{2}}{3}\;-\;\frac{h\Phi_{x}}{2}\;-\;\frac{\Phi h_{x}}{2}\;\right)\;y^{2}\right], \end{array}$$

(37) $$\begin{array}{cc} p^{\left(1\right)} & =-\frac{3h_{x}}{Re}\left(h+y\right), \end{array}$$

(38) $$\begin{array}{cc} T^{\left(1\right)} & =\frac{PeBi}{{\left(1+Bih\right)}^{2}}\times \\ & \left[\;-\frac{Bih_{x}}{20}y^{5}\;+\;\left(\frac{h_{x}}{12}\;+\;\frac{Bihh_{x}}{4}\right)y^{4}\;-\;\frac{Bih^{2}h_{x}}{3}y^{3}\;+\;\left(\frac{Bih^{4}h_{x}}{4}\;-\;\frac{h^{3}h_{x}}{3}\right)y\right], \end{array}$$

where

(39) $$\Phi =\frac{2\cot\beta }{Re}h_{x}-Wh_{xxx}.$$

Note that the temperature is not included in the solutions of velocity fields, and this result can be corrected by using the temperature-dependent physical properties such as viscosity, density, and surface tension. Finally, the kinematic equation for the free surface up to O(ϵ) can be expressed by

(40) $$h_{t}=-{\left(\frac{2}{3}h^{3}\right)}_{x}+\epsilon {\left(-\frac{8}{15}Reh^{6}h_{x}+\frac{2\cot\beta }{3}h^{3}h_{x}-\frac{1}{3}ReWh^{3}h_{xxx}\right)}_{x}.$$

The first term on the right-hand side of Equation (40) indicates the wave propagation. The second, third, and fourth terms represent the effects of the mean flow, hydrostatic pressure, and surface tension, respectively.

### 2.3. Linear Stability

When η is an infinitesimal disturbance against an initial flat film, it can be written as

(41) h=1+δη,

where δ is the initial amplitude of the disturbance. Inserting Equation (41) into Equation (40) and linearizing in δ up to O(δ) gives

(42) $$\eta_{t}=-2\eta_{x}+\epsilon \left(-\frac{8}{15}Re\eta_{xx}+\frac{2\cot\beta }{3}\eta_{xx}-\frac{1}{3}ReW\eta_{xxxx}\right)+O\left(\delta^{2}\right).$$

Substituting η=exp[i(αx−ct)] as the normal mode analysis into Equation (42) yields

(43) $$c=2\alpha +i\epsilon \alpha^{2}\left[\frac{8}{15}Re-\frac{2\cot\beta }{3}-\frac{1}{3}ReW\alpha^{2}\right],$$

where α is the scaled wavenumber and *c* is the complex number indicating wave frequency. Since the imaginary part of *c* represents the effective growth rate, Gr,

(44) $$Gr=\alpha^{2}\left[\frac{8}{15}Re-\frac{2\cot\beta }{3}-\frac{1}{3}ReW\alpha^{2}\right].$$

When Gr is a positive value, the amplitude of disturbance increases exponentially with time and instability occurs. Note that the real part of *c* indicates a phase speed of waves and Re(c)=2α.

### 2.4. Entropy Production Rate

The local entropy generation normalized by $k\Delta T^{2}/h_{N}^{2}$ can be written by

(45) $$s_{gen}=\epsilon^{2}T_{x}^{2}+T_{y}^{2}+V_{D}\left[2\epsilon^{2}\left(u_{x}^{2}+v_{y}^{2}\right)+{\left(\epsilon^{2}v_{x}+u_{y}\right)}^{2}\right],$$

where the viscous dissipation parameter $V_{D}$ is denoted as

(46) $$V_{D}=\frac{\mu U^{2}}{k\Delta T^{2}}.$$

The volumetric averaged entropy generation can be recast into

(47) $$S_{gen}=\frac{\int_{0}^{L}\int_{0}^{h}s_{gen}dydx}{\int_{0}^{L}\int_{0}^{h}dydx}=N_{1}+N_{2}+N_{3},$$

where

(48) $$\begin{array}{cc} N_{1} & =\frac{\epsilon^{2}}{A}\int_{0}^{L}\int_{0}^{h}T_{x}^{2}dydx∼\frac{\epsilon^{2}}{A}\int_{0}^{L}\left[\frac{Bi^{4}}{3{\left(1+Bih\right)}^{4}}h^{3}h_{x}^{2}\right]dx+O\left(\epsilon^{3}\right), \end{array}$$

(49) $$\begin{array}{ccc} N_{2} & = & \frac{1}{A}\int_{0}^{L}\int_{0}^{h}T_{y}^{2}dydx\\ & = & \frac{1}{A}\int_{0}^{L}\left[\frac{Bi^{2}h}{{\left(1+Bih\right)}^{2}}+\epsilon \frac{PeBi^{2}h^{4}h_{x}}{{\left(1+Bih\right)}^{4}}\left(\frac{1}{2}+\frac{4}{15}Bih-\frac{7}{30}Bi^{2}h^{2}\right)\right.\\ & & \;+\left.\epsilon^{2}\frac{Pe^{2}Bi^{2}h^{7}h_{x}^{2}}{{\left(1+Bih\right)}^{4}}\left(\frac{1}{14}-\frac{367}{5040}Bih+\frac{107}{5040}Bi^{2}h^{2}\right)\right]dx\;+\;O\left(\epsilon^{3}\right), \end{array}$$

(50) $$\begin{array}{cc} N_{3} & =\frac{V_{D}}{A}\int_{0}^{L}\int_{0}^{h}\left[2\epsilon^{2}\left(u_{x}^{2}+v_{y}^{2}\right)+{\left(\epsilon^{2}v_{x}+u_{y}\right)}^{2}\right]dydx\\ & ∼\frac{V_{D}}{A}\int_{0}^{L}\left\{\frac{4}{3}h^{3}+\epsilon Re\left(\frac{32}{15}h_{x}h^{6}-\frac{4}{3}\Phi h^{3}\right)\right.\\ & \;+\;\left.\epsilon^{2}\;\left[-\frac{1}{3}h^{4}h_{xx}\;+\;\frac{16}{3}h^{3}h_{x}^{2}\;+\;Re^{2}h^{3}\;\left(\frac{272}{315}h^{6}h_{x}^{2}\;-\;\frac{16}{15}\Phi h_{x}h^{3}\;+\;\frac{1}{3}\Phi^{2}\;\right)\;\right]\;\right\}dx\\ & \;+O(\epsilon^{3}), \end{array}$$

where $A=\int_{0}^{L}hdx$ is a volume enclosed between the free surface and the *x*-axis with period *L* per unit spanwidth. The derivations of Equations (48)–(50) are summarized in Appendix A. All terms of Equation (47) are expanded in the order of ϵ and are arranged up to $O(\epsilon^{2})$. Note that $N_{1}$ and $N_{2}$ denote the volumetric averaged entropy produced by heat transfer in the lateral direction and in the transverse direction, respectively, and $N_{3}$ represents the volumetric averaged entropy generated by the fluid friction.

When the flat free surface is considered, the solutions of the leading order with h=1 can be summarized as

(51) $$\begin{array}{c} u^{\left(0\right)}=2\left(y-\frac{y^{2}}{2}\right)\;\;\;\mathrm{and}\;\;\;v^{\left(0\right)}=0. \end{array}$$

Then, $N_{3}$ with the flat free surface can be reduced to

(52) $$N_{3}=\frac{V_{D}}{A}\int_{0}^{L}\int_{0}^{h}{\left(u^{\left(0\right)}\right)}_{y}^{2}dydx=V_{D}\int_{0}^{1}\left(1-2y+y^{2}\right)dy=\frac{4}{3}V_{D}.$$

This result can be easily checked from Equation (50), neglecting all terms including the derivatives of *x*.

Finally the irreversibility ratio Ψ is defined by

(53) $$\Psi =\frac{N_{3}}{N_{1}+N_{2}}.$$

When the viscous dissipation parameter increases, the magnitude of $N_{3}$ and the irreversibility ratio Ψ are also increased. Note that the irreversibility ratio denotes the relative magnitude of entropy generation originated by fluid friction and heat transfer. When Ψ>1, the fluid friction gives much contribution to entropy production more than heat transfer.

## 3. Results

In this study, the falling film with an inclination angle β=π/4 and with period of L=2π is considered for the generation of entropy with time. The parameters of Pe=1 and Bi=10 were used for temperature fields, unless otherwise specified. From Equation (44) as the results of the linear stability analysis, both unstable and stable conditions with α=1 were considered: (1) Gr>0 with Re=10 and W=1 and (2) Gr<0 with Re=1 and W=5. In order to compute velocity and temperature fields with time, it is necessary to obtain the evolution of film depth *h*. First, the evolution of the film height for Equation (40) with periodic conditions was solved by employing the method of lines (MOL), where the right hand side of Equation (40) with ϵ=1/L is computed by FFT (Fast Fourier transform) method. The resultant ordinary differential equation (ODE) is computed by means of the MATLAB integrator ode113.m with tolerance of $10^{-13}$, where ODE113 is a solver to use a multistep method with an algorithm of the Adams–Bashforth–Moulton method for ODE. The initial condition for *h* is assumed by

(54) h(x,0)=1+0.1cos(x).

The velocity and temperature fields for Equations (35), (36) and (38) can be obtained from the solutions of *h* and its derivatives, where the derivatives of *h* are calculated by FFT based on each solution of *h* at time *t*. Finally, the volumetric averaged entropy productions of $N_{1}$, $N_{2}$, and $N_{3}$ are numerically calculated by the Simpson rule.

**Figure 2:**
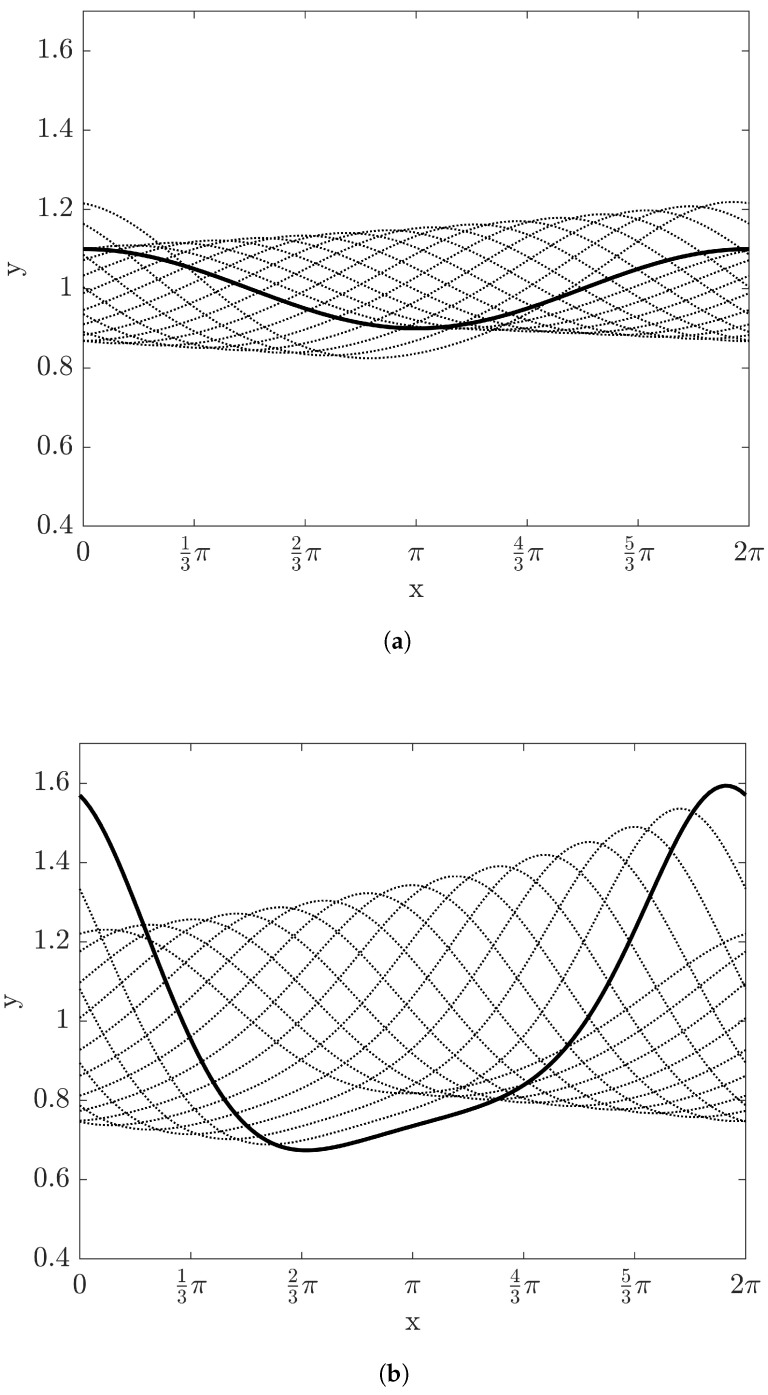
The evolution of film height with time with Re=10, β=π/4, and W=1: (**a**) 0<t<3 and (**b**) 3.2<t<6. The time step between the curves is 0.2. Solid thick curves of (**a**,**b**) represent the film height at t=0 and at t=6.

The evolution of film depth with time up to t=6 is shown in Figure 2 with Re=10 and W=1, where each curve is overlapped with Δt=0.2. As time elapses, the amplitude of film height increases and the evolution of the film shows instability with the expectation by the linear stability analysis. Since the crest of the film moves faster than the trough of the film, the film with this instability will experience a wave breaking.

**Figure 3:**
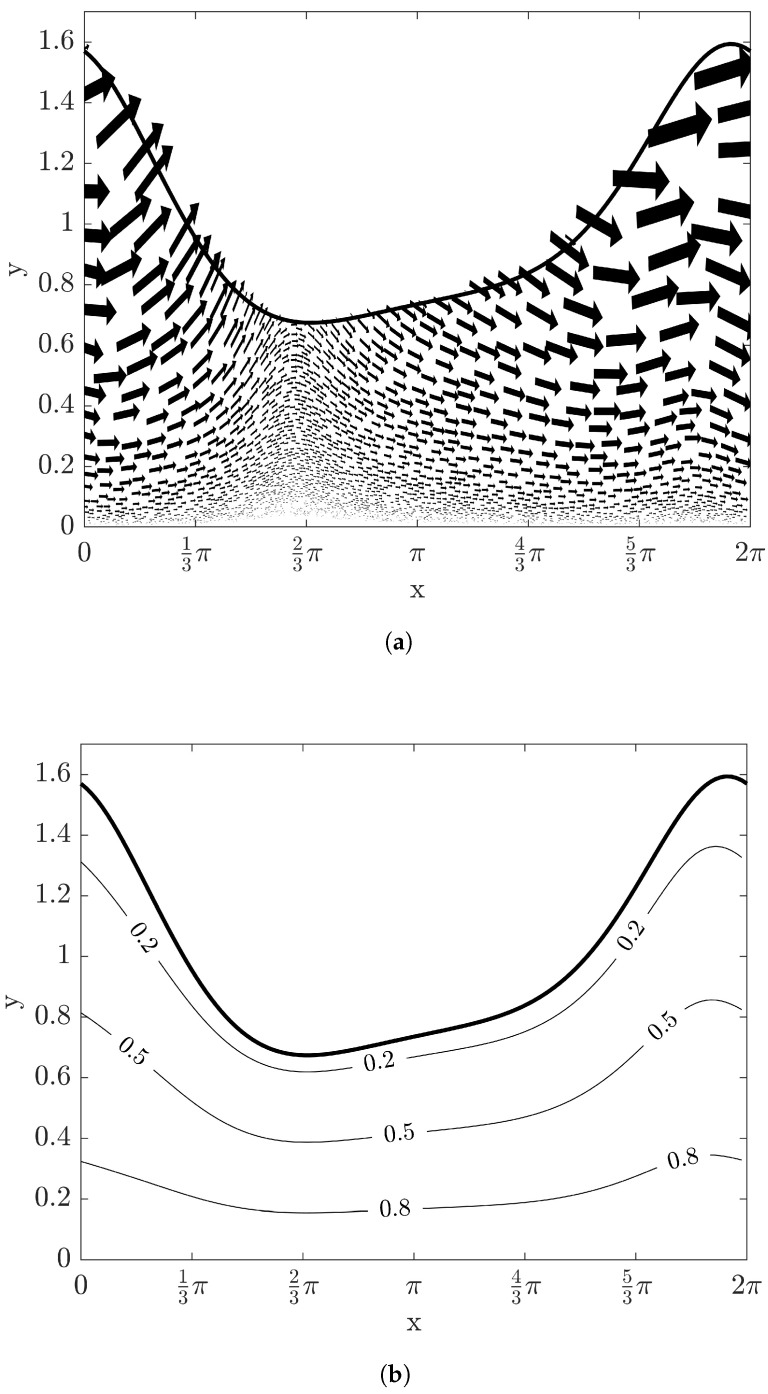
The vector and contour plots at time t=6 with Re=10, β=π/4, and W=1: (**a**) velocity fields and (**b**) temperature fields. The solid thick curve represents the film height at t=6.

Figure 3a with the same condition of Figure 2 shows the velocity fields in a vector plot. Here, the velocity fields from Equations (22), (23), (35) and (36) are not affected by the temperature fields due to constant physical properties such as viscosity and density. Temperature distribution with Bi=10 within the film is represented in Figure 3b. In case of the adiabatic condition of Bi→0, it can be easily verified that T=1 within the film flowing down an inclined plane at quasi-steady state from Equations (25) and (38). When heat removes effectively through the interface to the ambient phase (i.e., Bi→∞), the temperature distribution can be rearranged as

(55) $$\begin{array}{cc} T(x,y,t) & =1-\frac{y}{h}+\epsilon \frac{Pe}{h^{2}}\left[-\frac{h_{x}}{20}y^{5}\;+\;\frac{hh_{x}}{4}y^{4}\;-\;\frac{h^{2}h_{x}}{3}y^{3}\;+\;\frac{h^{4}h_{x}}{4}y\right]. \end{array}$$

The temperature at the interface is reduced into $T=7\epsilon Peh_{x}h^{3}/60$ from Equation (55) and decreases as Pe decreases.

**Figure 4:**
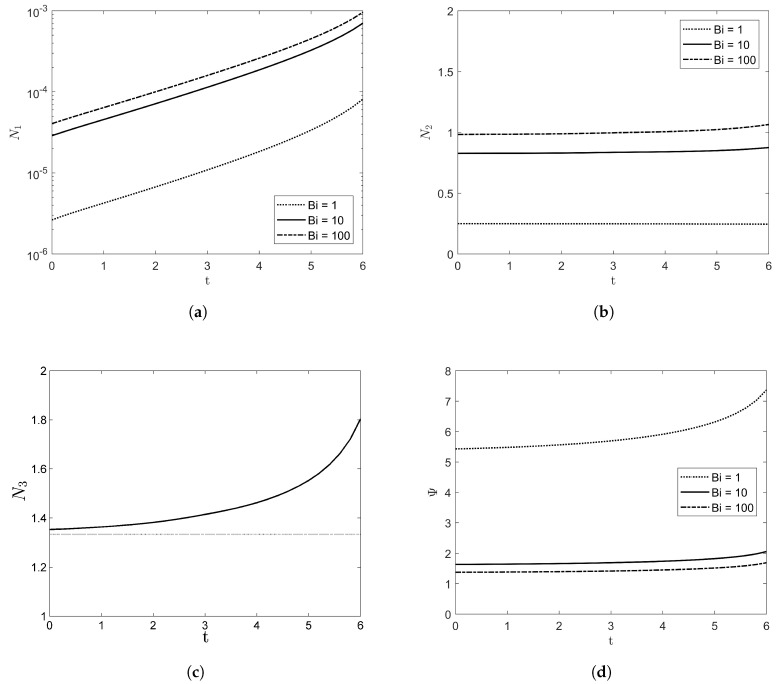
The entropy production rate of (**a**) $N_{1}$, (**b**) $N_{2}$, (**c**) $N_{3}$, and (**d**) Ψ with Re=10, β=π/4, W=1, and $V_{D}=1$.

Figure 4 shows the volumetric averaged entropy production rate of $N_{1}$, $N_{2}$, and $N_{3}$ with $V_{D}=1$. As the amplitude of the film grows, it can be easily verified that entropy production increases. While $N_{1}$ is exponentially increased as time goes, the magnitude of $N_{1}$ is much less than those of $N_{2}$ and $N_{3}$. As Bi goes to infinity, the entropy production by heat transfer (i.e., both of $N_{1}$ and $N_{2}$) increases, but the irreversibility ratio Ψ decreases as illustrated in Figure 4d. Note that both of $N_{1}$ and $N_{2}$ will be zero as Bi→0 from Equations (48) and (49) and $N_{3}$ does not depend on Bi from Equation (50) due to constant physical properties. As Bi→∞, the entropy production of $N_{1}$ and $N_{2}$ can be reduced as

(56) $$\begin{array}{c} \begin{array}{cc} N_{1} & ∼\frac{\epsilon^{2}}{A}\int_{0}^{L}\left[\frac{h_{x}^{2}}{3h}\right]dx+O\left(\epsilon^{3}\right), \end{array} \end{array}$$

(57) $$\begin{array}{c} \begin{array}{cc} N_{2} & ∼\frac{1}{A}\int_{0}^{L}\left[\frac{1}{h}-\epsilon \frac{7}{30}Peh^{2}+\epsilon^{2}\frac{107}{5040}Pe^{2}h^{5}h_{x}^{2}\right]+O\left(\epsilon^{3}\right), \end{array} \end{array}$$

The entropy production with the deformable free surface is compared with that of the flat free surface in Figure 4c, where the dotted line denotes the entropy production with the non-deformable free surface. From Equation (52) with $V_{D}=1$, it can be verified that $N_{3}=4/3$.

**Figure 5:**
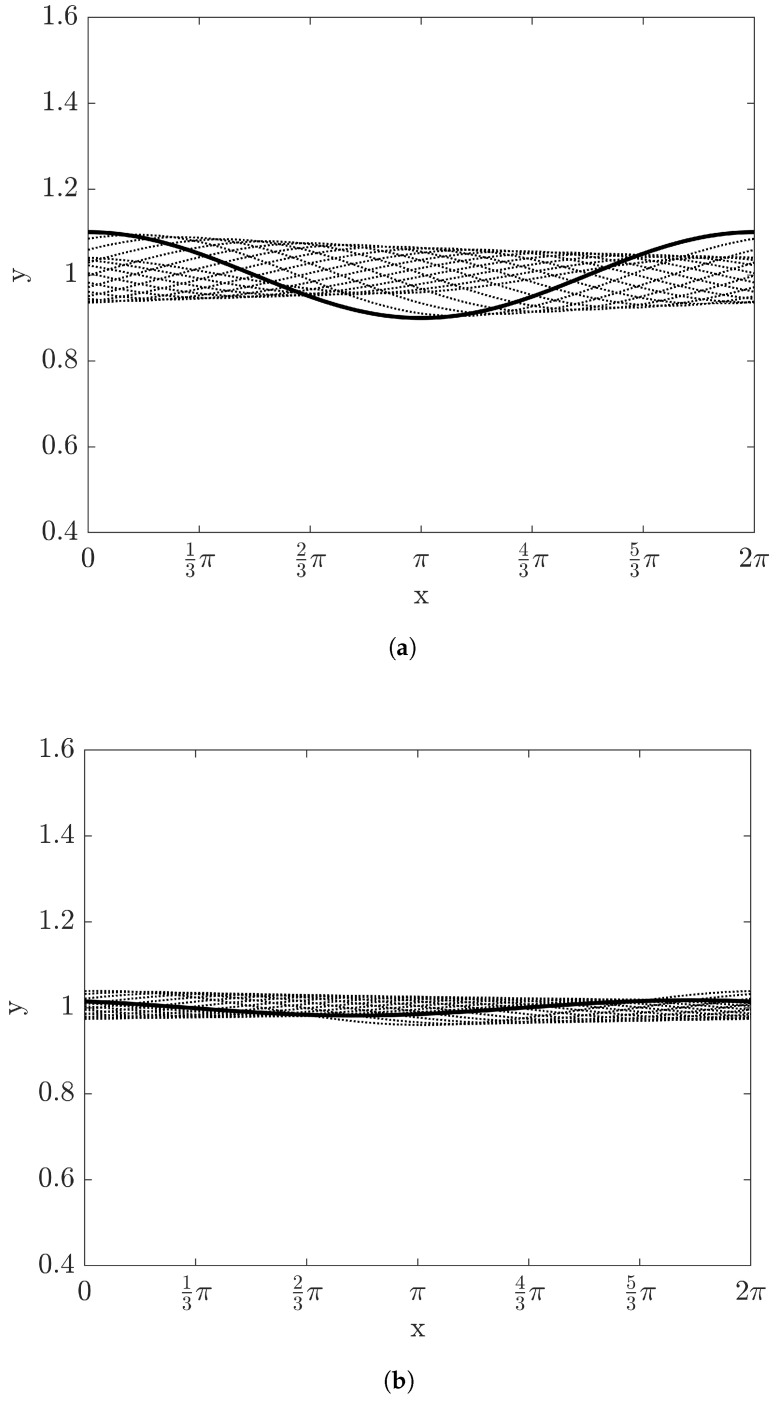
The evolution of film height with time with Re=1, β=π/4, and W=5: (**a**) 0<t<3 and (**b**) 3.2<t<6. The time step between the curves is 0.2. The solid thick curves of (**a**,**b**) represent the film height at t=0 and at t=6, respectively.

**Figure 6:**
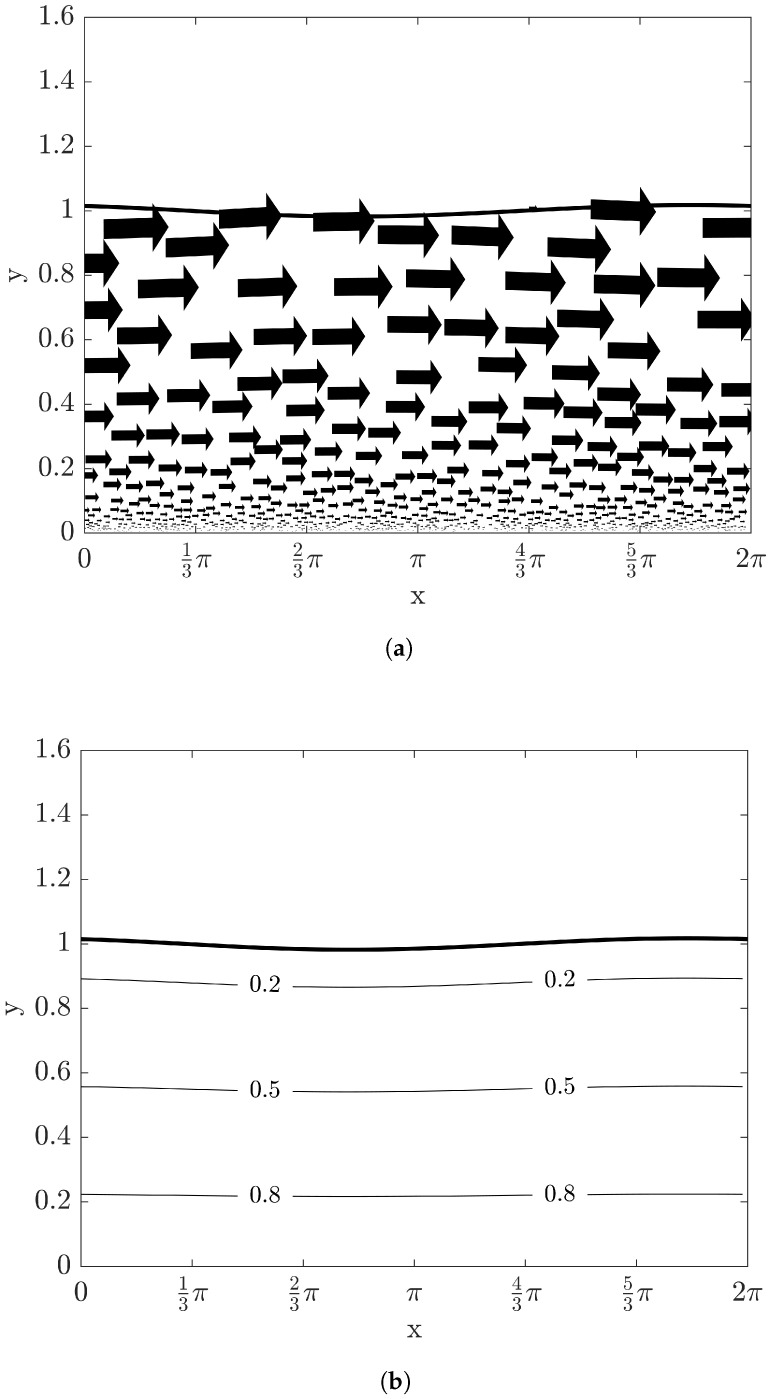
The vector and contour plots at time t=6 with Re=1, β=π/4, and W=5: (**a**) velocity fields and (**b**) temperature fields. The solid thick curve represents the film height at t=6.

**Figure 7:**
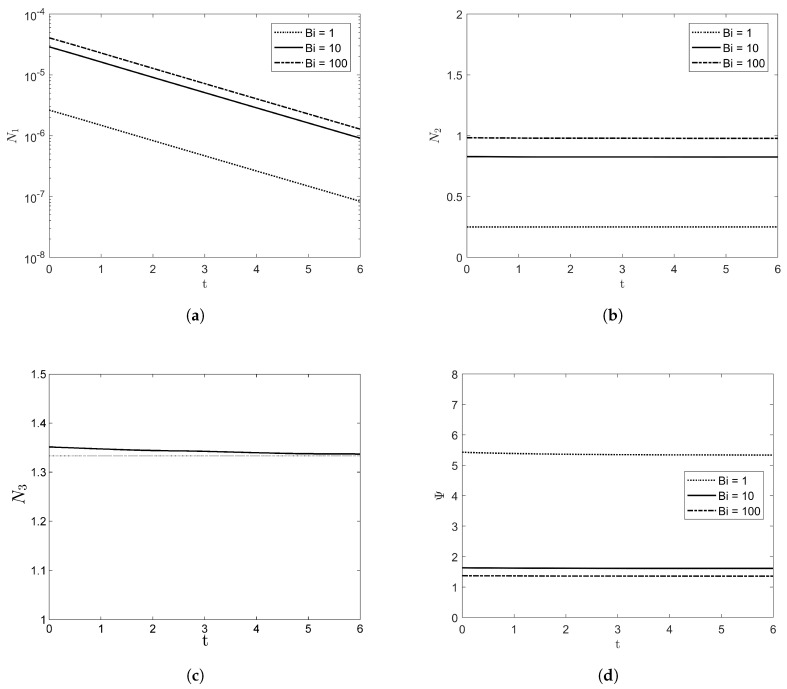
The entropy production rate of (**a**) $N_{1}$, (**b**) $N_{2}$, (**c**) $N_{3}$, and (**d**) Ψ with Re=1, β=π/4, W=5, and $V_{D}=1$.

In the case of stable film flow with Re=1 and W=5, the evolution of film depth with time up to t=6 is shown in Figure 5, where each curve is overlapped with Δt=0.2. As time grows, the amplitude of film height decreases and asymptotically approaches flat. The velocity and temperature fields are illustrated in Figure 6. The vector plot shows that the flow direction is nearly parallel to the *x*-axis and the contour line of temperature fields are also almost flat. These stable patterns make the entropy production in the lateral direction reduce significantly, as shown in Figure 7. The variations of $N_{2}$ and $N_{3}$ are negligible and $N_{3}$ is asymptotically reduced to the value with the flat interface. Therefore, the value of the irreversibility ratio is nearly constant depending on Bi.

**Figure 8:**
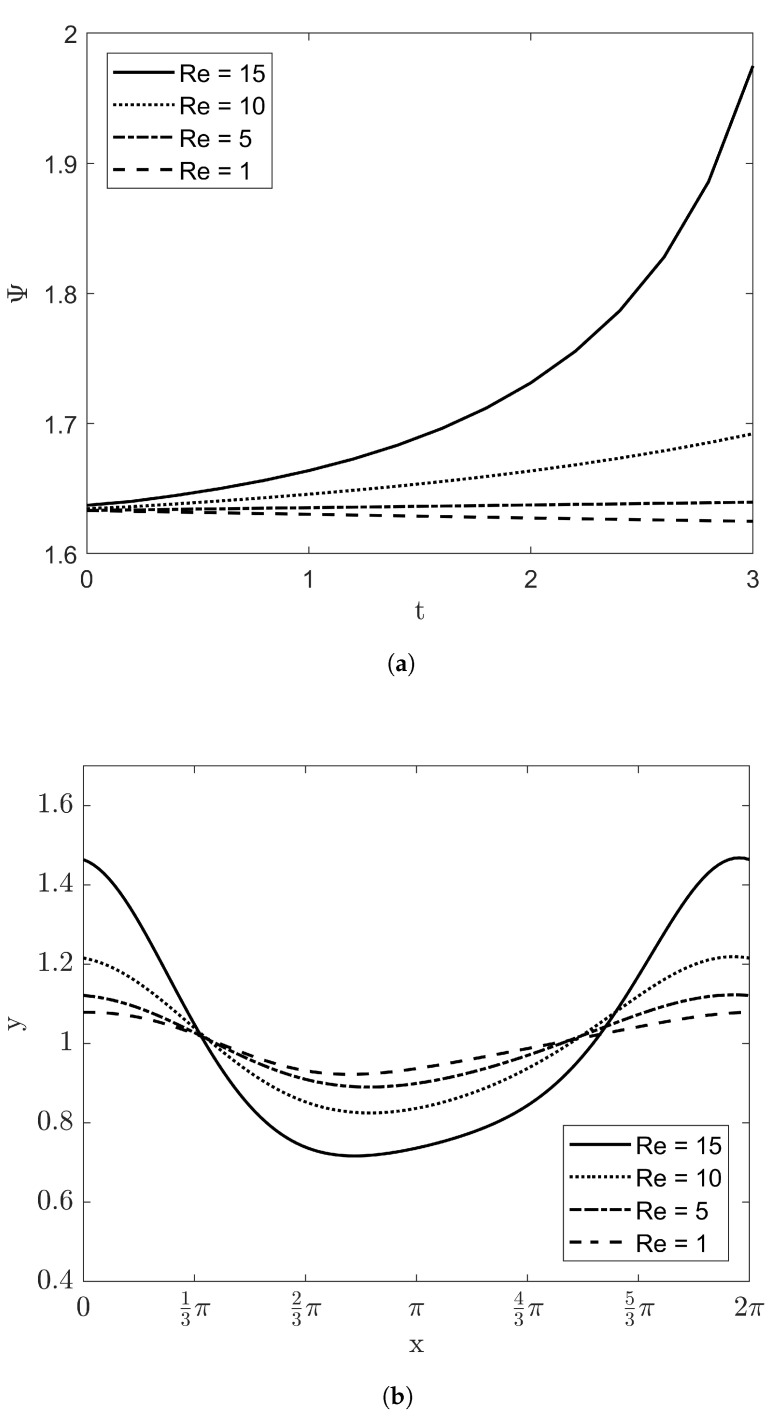
The irreversibility ratio and the evolution of film thickness with $V_{D}=1$, β=π/4, Bi=10, and W=1: (**a**) Ψ within 0<t<3 and (**b**) the film height at t=3.

**Figure 9:**
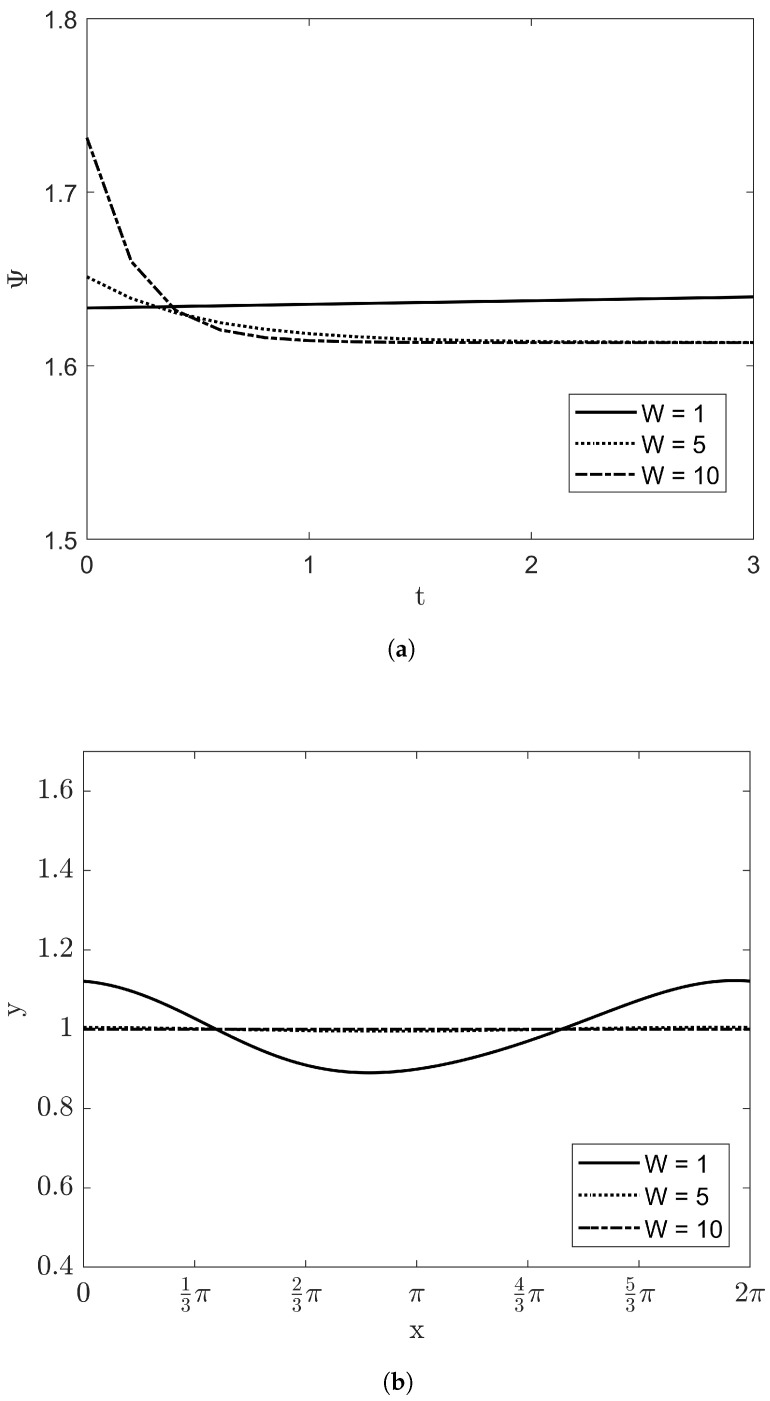
The irreversibility ratio and the evolution of film thickness with $V_{D}=1$, β=π/4, Bi=10, and Re=5: (**a**) Ψ within 0<t<3 and (**b**) the film height at t=3.

The parametric studies of Re and *W* were carried out with conditions of $V_{D}=1$, β=π/4, and Bi=10. Figure 8a illustrates the irreversibility ratio Ψ with Reynolds number. As the Reynolds number increases, the value of Ψ is also increasing with time. The shape of film height at t=3 is displayed at Figure 8b. The amplitude of film height at high Reynolds number is much larger than that of film thickness at low Reynolds number. These results are coincident with the linear stability analysis. Above Re>5, the growth rate Gr from Equation (44) is larger than zero. Figure 9 demonstrates the effects of *W* at fixed Reynolds number Re=5. When W=1, it can be verified that the irreversibility ratio and the amplitude of the film thickness increase slightly with time. As *W* as the stabilizing factor grows, the dynamics of the film flow makes weak and reduces into the flat film.

## 4. Discussion

The entropy production on a gravity-driven falling film along an inclined heated plane was obtained by the long-wave approach. Here, the velocity and temperature fields were decided by the evolution equation from the kinematic condition at the free interface. The instability of the flowing film makes the entropy production increase because the gradient of velocity fields in the liquid layer becomes large. The present work shows that Bi has an important role in the entropy production due to heat transfer. In the case of adiabatic conditions (i.e., Bi goes to zero), the liquid layer has the same temperature due to constant wall temperature condition. As Bi increases, the temperature variation inside flowing films depends on Bi and this affects the entropy generation by heat transfer of $N_{1}$ and $N_{3}$. The value of the irreversibility ratio also shows dependency on Bi. The contribution of fluid friction on the volumetric averaged entropy production is larger than that of heat transfer at moderate and high viscous dissipation parameters.

The entropy production by fluid friction is not affected by heat transfer. This result is due to constant physical properties such as density, viscosity, and surface tension. As an additional future work, variable viscosity and surface tension will be included in next topics and the case of constant heat flux will also be investigated.

## Nomenclature

*c*	complex number indicating wave frequency
$C_{p}$	heat capacity
*g*	gravitational acceleration constant
Gr	effective growth rate
$h_{N}$	Nusselt fiat film thickness
$h_{T}$	heat transfer coefficient of the ambient phase
*k*	liquid thermal conductivity
*L*	characteristic length
$N_{1}$ and $N_{2}$	volumetric averaged entropy generation rate of heat transfer in the lateral and transverse directions
$N_{3}$	volumetric averaged entropy generation rate by fluid friction
*p* and $p_{0}$	pressure in liquid and gas phases
$s_{gen}$	local entropy generation rate
$S_{gen}$	volumetric averaged entropy generation rate
*T* and $T_{0}$	temperature in liquid and ambient phases
$T_{B}$	bottom temperature
*U*	maximum velocity of the Nusselt film
$V_{D}$	viscous dissipation parameter defined by $\mu U^{2}/k\Delta T^{2}$
u=(u,v)	velocity fields in liquid phase
Re	Reynolds number defined by $Uh_{N}/\nu$
Fr	Froude number defined by $U/\sqrt{gh_{N}}$
Pe	Peclet number defined by $Uh_{N}/\kappa$
Bi	Biot number defined by $h_{T}h_{N}/k$
*W* and We	the modified Weber number ($\epsilon^{2}We$) and Weber number defined by $\sigma /\rho U^{2}h_{N}$
α	scaled wavenumber
β	inclination angle
δ	initial amplitude of the disturbance
ϵ	long-wave parameter defined by $h_{N}/L$
κ	thermal diffusivity defined by $k/\rho C_{p}$
η	infinitesimal disturbance against an initial flat film
ν	kinematic viscosity
ρ	liquid density
μ	liquid viscosity
Ψ	irreversibility ratio defined by $N_{3}/\left(N_{1}+N_{2}\right)$
σ	surface tension

## Appendix A. Derivations of Equations (48)–(50)

Since $T=T^{\left(0\right)}+\epsilon T^{\left(1\right)}$ with Equations (22), (23) and (38), $T_{x}$ can be easily computed by

(A1) $$T_{x}=\frac{Bi^{2}}{{\left(1+Bih\right)}^{2}}h_{x}y+O\left(\epsilon \right).$$

Then, the first term $N_{1}$ up to $O(\epsilon^{3})$ is given by

(A2) $$\begin{array}{c} N_{1}=\epsilon^{2}\int_{0}^{L}\int_{0}^{h}T_{x}^{2}dydx=\epsilon^{2}\int_{0}^{L}\frac{Bi^{4}}{3{\left(1+Bih\right)}^{4}}h^{3}h_{x}^{2}dx+O\left(\epsilon^{3}\right). \end{array}$$

$T_{y}$ can be also summarized as

(A3) $$\begin{array}{cc} T_{y} & =-\frac{Bi}{1+Bih}+\frac{PeBi}{{\left(1+Bih\right)}^{2}}\times \\ & \left[-\frac{Bih_{x}}{4}y^{4}+\left(\frac{h_{x}}{3}+Bihh_{x}\right)y^{3}-Bih^{2}h_{x}y^{2}+\frac{Bih^{4}h_{x}}{4}-\frac{h^{3}h_{x}}{3}\right]. \end{array}$$

Substituting Equation (A3) into Equation (49) and arranging gives

(A4) $$\begin{array}{cc} N_{2} & =\int_{0}^{L}\int_{0}^{h}T_{y}^{2}dydx\\ & =\int_{0}^{L}\left[\frac{Bi^{2}h}{{\left(1+Bih\right)}^{2}}+\epsilon \frac{PeBi^{2}h^{4}h_{x}}{{\left(1+Bih\right)}^{4}}\left(\frac{1}{2}+\frac{4}{15}Bih-\frac{7}{30}Bi^{2}h^{2}\right)\right.\\ & \;+\left.\epsilon^{2}\frac{Pe^{2}h^{7}h_{x}^{2}}{{\left(1+Bih\right)}^{4}}\left(\frac{1}{14}-\frac{367}{5040}Bih+\frac{107}{5040}Bi^{2}h^{2}\right)\right]dx+O\left(\epsilon^{3}\right). \end{array}$$

Since $u=u^{\left(0\right)}+\epsilon u^{\left(1\right)}$ with Equations (22), (23), (35) and (36), $N_{3}$ from Equation (50) with the incompressibility condition (i.e., $u_{x}^{2}+v_{y}^{2}={\left(u_{x}+v_{y}\right)}^{2}-2u_{x}v_{y}=-2u_{x}v_{y}$) can be recast into

(A5) $$\begin{array}{cc} N_{3} & =\int_{0}^{h}\int_{0}^{L}\left[2\epsilon^{2}\left(u_{x}^{2}+v_{y}^{2}\right)+{\left(\epsilon^{2}v_{x}+u_{y}\right)}^{2}\right]dxdy\\ & =\int_{0}^{h}\int_{0}^{L}\left[-4\epsilon^{2}u_{x}v_{y}+{\left(\epsilon^{2}v_{x}+u_{y}\right)}^{2}\right]dxdy\\ & ∼\int_{0}^{L}\left\{\frac{4}{3}h^{3}+\epsilon Re\left(\frac{32}{15}h_{x}h^{6}-\frac{4}{3}\Phi h^{3}\right)\right.\\ & \left.+\epsilon^{2}\;\left[\;-\frac{1}{3}h^{4}h_{xx}\;+\;\frac{16}{3}h^{3}h_{x}^{2}\;+\;Re^{2}h^{3}\left(\frac{272}{315}h^{6}h_{x}^{2}\;-\;\frac{16}{15}\Phi h_{x}h^{3}\;+\;\frac{1}{3}\Phi^{2}\;\right)\;\right]\;\right\}\;dx\\ & \;+O(\epsilon^{3}), \end{array}$$

where

(A6) $$\begin{array}{cc} u_{x} & =2h_{x}y+\epsilon Re\left(\;\frac{y^{4}}{6}\left(h_{x}^{2}+hh_{xx}\right)-\frac{y^{3}}{3}\left(4hh_{x}^{2}+2h^{2}h_{xx}\right)+\frac{y^{2}}{2}\Phi_{x}\right.\\ & \;\;\;\;\;\;\;\left.+\frac{y}{3}\left(16h^{3}h_{x}^{2}\;+\;4h^{4}h_{xx}\right)-\left(\Phi_{x}h+\Phi h_{x}\right)y\right), \end{array}$$

(A7) $$\begin{array}{cc} u_{y} & =2\left(h-y\right)+\epsilon Re\left(\frac{2}{3}hh_{x}^{2}y^{3}-2h^{2}h_{x}y^{2}+\Phi y+\frac{4h^{4}h_{x}}{3}-\Phi h\right), \end{array}$$

(A8) $$\begin{array}{cc} v_{x} & =\;-h_{xx}y^{2}\;\\ & +\;\epsilon Re\left[-\left(hh_{xxx}\;+\;3h_{x}h_{xx}\right)\frac{y^{5}}{30}\;+\;\left(6hh_{x}h_{xxx}\;+\;h^{2}h_{xxx}\;+\;2h_{x}^{3}\right)\frac{y^{4}}{6}\;-\;\frac{\Phi_{xx}}{6}y^{3}\right.\\ & \;-\left.\left(\;8h^{3}h_{x}h_{xx}\;+\;\frac{2}{3}h^{4}h_{xxx}\;+\;8h^{2}h_{x}^{3}\;-\;h_{x}\Phi_{x}\;-\;\frac{1}{2}h\Phi_{xx}\;+\;\frac{1}{2}\Phi h_{xx}\right)\;y^{2}\right], \end{array}$$

(A9) $$\begin{array}{cc} v_{y} & =-2h_{x}y+\epsilon Re\;\left[\;-\;\left(\;\frac{hh_{xx}}{6}\;+\;\frac{h_{x}^{2}}{6}\;\right)\;y^{4}+4\left(\;\frac{h^{2}h_{xx}}{6}\;+\;\frac{hh_{x}^{2}}{3}\;\right)\;y^{3}\right.\\ & \;\;\;\;\;\;\;\;\left.\;-\frac{\Phi_{x}}{2}y^{2}\;-\;\left(\;\frac{4h^{4}h_{xx}}{3}\;+\;\frac{16h^{3}h_{x}^{2}}{3}\;-\;h\Phi_{x}\;-\;\Phi h_{x}\;\right)\;y\right]. \end{array}$$
